# Fracture Characteristics and Analysis in Dissimilar Cu-Al Alloy Joints Formed via Electromagnetic Pulse Welding

**DOI:** 10.3390/ma12203368

**Published:** 2019-10-15

**Authors:** Puquan Wang, Daolun Chen, Yang Ran, Yunqi Yan, He Peng, Xianquan Jiang

**Affiliations:** 1 College of Engineering and Technology, Southwest University, Tiansheng Road 2, Beibei District, Chongqing 400715, China; 13698250260@163.com (P.W.); penghe@swu.edu.cn (H.P.); 2 Department of Mechanical and Industrial Engineering, Ryerson University, 350 Victoria Street, Toronto, ON M5B 2K3, Canada; 3 Institute of Optics and Mechanics, Chongqing Academy of Science and Technology, Yangliu Road 2, Chongqing 401123, China; forestchn@aliyun.com; 4 Department of Mechanical and Engineering, Sichuan University of Science and Engineering, Huixing Road 519, Zigong 643000, China; 15808204693@163.com; 5 College of Materials and Energy, Southwest University, Tiansheng Road 2, Beibei District, Chongqing 400715, China

**Keywords:** electromagnetic pulse welding, aluminum alloy, copper, fatigue fracture, finite element simulation

## Abstract

The aim of this study was to identify and analyze the fatigue fracture characteristics of dissimilar Al 6061 to Cu (UNS C11000) lap joints made with ultrafast electromagnetic pulse welding (EMPW) via fractography, stress analysis and finite element simulation. It was observed that EMPW generated an annular (or ring-shaped) bonding area, with weld zones and a central non-weld zone when viewed from the cross section. Two types of failure modes occurred in relation to the cyclic loading levels: base metal fracture or transverse through-thickness (TTT) crack growth at a higher loading level, and joint interfacial failure at a lower loading level. In the interfacial failure, fatigue crack initiated from the outer edge of annular welding area, and propagated to form an approximate elliptical boundary. Fatigue crack propagation was characterized by fatigue striations existing in discrete areas on the fracture surface. This was attributed to a coupled role of shear and normal stresses present in a tensile lap shear sample due to the bending moment caused by the inherent misalignment. The final rapid fracture started from elliptical boundary with elongated shear dimples. Both theoretical stress analysis and finite element model revealed the maximum stress and stress concentration along the outer edge, where fatigue crack initiation occurred.

## 1. Introduction

In recent years, vehicle lightweighting has played a key part in energy saving and environment protection in the transportation industry [1,2,3,4]. Aluminum alloys, as an important type of lightweight material, have become increasingly popular due to their high strength-to-weight ratio, good formability, machinability and recyclability [5]. Meanwhile, there is a high demand to weld Al to other materials, namely, Fe, Cu, Mg, plastics, etc., for maximizing the potential of lightweight structures and electrical power components (e.g., power cables, connectors and terminals) associated with relatively low weight and cost in electrical vehicles [6,7]. However, this is difficult to accomplish using fusion processes due to the different melting points and physical properties of dissimilar materials. Accordingly, developing solid-state joining techniques is the key to expanding the use of dissimilar joints of Al to other materials in the automotive industry [8,9,10].

Solid-state joining, defined as a joining process without any liquid or vapor phase, involves the use of pressure and kinetic (or thermal) energy to achieve joining via appreciable deformation and solid-state diffusion, including friction stir welding [11,12], ultrasonic spot welding [13,14], linear friction welding [15,16], etc. High-speed impact welding techniques, such as electromagnetic pulse welding (EMPW) and explosive welding (EXW), share the same welding mechanism, but have different welding energy sources as indicated by their names, specifically capacitor bank energy (MPW) [17] and explosions [18], respectively. While EXW can be used in producing large components, it is not easy to control the magnitude of energy, and is not well suited for automotive applications. Thus the environmental-friendly, highly-efficient and easy-automated EMPW technique has attracted increasing attention of the manufacturing industry and researchers because of the capability of joining dissimilar materials with good stability and quality without heat energy input. Additionally, the whole process of EMPW just needs 40~60 µs, which would limit the formation of undesirable compounds and metallurgical defects that are commonly observed in most other fusion welding techniques [19]. Therefore, EMPW represents a promising alternative for the automotive industry.

The automotive applications of dissimilar welded joints inevitably involve safety and structural integrity, which can be improved by increasing the interfacial bonding strength. Some recent studies on failure mode and fracture behavior were reported, aimed at analyzing force state and plastic deformation features of welded joints. Geng et al. [20] performed quasi-static and dynamic tensile tests to investigate cleavage and ductile fracture of EMPWed Al-Fe joints, and observed that the strain rate sensitivity of the bonded area led to differential plastic deformation, which may affect the strength and ductility. In another work, Liu et al. [21] studied the dissimilar joints of AA6061-T4 to cast magnesium AM60B made with vaporizing foil actuator welding (VFAW), where the welding zone exhibited a similar annular (or ring-shaped) morphology to that of an EMPWed lap joint. The lap-shear failure occurred mainly in the base material and the majority of the peeled fracture surface was ductile in nature, showing the good joining strength and better performance of plastic deformation. Furthermore, Macwan et al. [22] reported three failure modes: interfacial failure, failure at some distance away from the nugget edge (also referred to as transverse through-thickness crack growth), and nugget edge failure in an USWed (Ultrasonic spot welded) dissimilar Al/steel lap joint. Although these studies reported some fracture surface characteristics of joints welded with different techniques, to the best of our knowledge, there is no study on the fracture characteristics of EMPWed Al-Cu dissimilar lap joints in the open literature. In addition, no information is available about crack initiation of EMPWed joints via finite element simulation. The present study was, therefore, aimed at gaining a better understanding of fracture mechanisms and characteristics of EMPWed dissimilar Al-to-Cu joints via theoretical stress calculation and finite element analysis.

## 2. Materials and Methods

**Figure 1:**
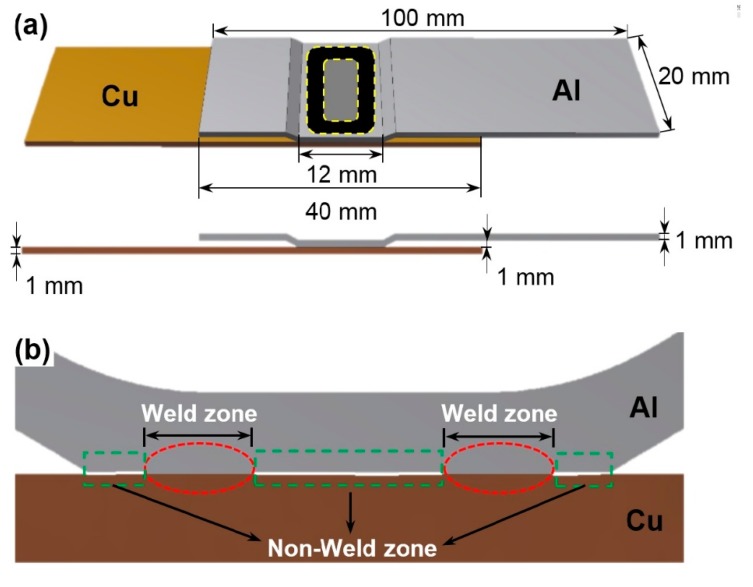
Schematic diagram of an Al-Cu lap shear test sample. (**a**) Welding location and sample dimensions; (**b**) enlarged view of the cross-section of Al-Cu lap joint, showing the weld zones and non-weld zones.

In the present study, EMPWed lap joints of 6061-O Al alloy sheets (100 mm × 20 mm × 1 mm) and oxygen-free pure (99.9 wt.%) copper-T2 (equivalent to UNS C11000) sheets (100 mm × 20 mm × 1 mm), with the detailed chemical compositions given in Table 1 and Table 2, were made with a Pulsar electromagnetic pulse welding machine of 70 kJ capacity (Chongqing Pulsa Technology Co. LTD, Chongqing, China) along with an E-shaped one-turn flat coil actuator covered by insulator, as schematically shown in Figure 1a. To have a flat and fresh surface, the edges of the cut sheets were deburred, cleaned with ethanol followed by acetone, and dried before welding. The EMPW was performed on a pair of Al/Cu sheets with an overlap of 40 mm (Figure 1a) and an initial gap of 1 mm between two sheets. The total length of welded samples was 160 mm. Figure 1b shows an enlarged view of the cross section of Al-Cu lap joint in Figure 1a, illustrating the weld zone and non-weld zone across an EMPWed lap joint. During welding, when the collision angle increased to a value large enough to form joint, the high-speed flying sheet started to hit and weld to the parent sheet, resulting in the formation of the weld zone and non-weld zone. The major EMPW parameters included discharge voltage (kV) and gap (mm) between flying and parent sheets, which affect collision angle and effective welding area directly [23]. In this study, Cu was selected as a parent sheet and Al as a flying sheet, as shown in Figure 1, and the welding parameters of V = 14 kV and g = 1 mm were chosen to make the Al/Cu dissimilar welded lap joints.

Load-control fatigue tests in accordance with ASTM E466-15 [24] on a fully computerized Instron 8801 servo-hydraulic testing system (Instron, Norwood, MA, USA) were conducted to investigate failure mode and fracture behavior of EMPWed Al/Cu dissimilar lap welded joints. To avoid potential buckling of the test specimens, tension-tension cyclic loading at a stress ratio of R (P_min_/P_max_) = 0.2 was applied at a frequency of 50 Hz with a sinusoidal waveform. The fracture surface was examined via a scanning electron microscopy (SEM, JEOL JSM-6480LV, JEOL, Tokyo, Japan) equipped with energy-dispersive X-ray spectroscopy (EDS). Theoretical calculations of forces based on the actual loading and the fundamentals of mechanics of materials were implemented to figure out the overall force condition of lap welded joints with an annular bonding area. In addition, finite element simulation of Al/Cu lap joints during fatigue was also carried out via ABAQUS software (version 6.13, HKS, Atlanta, GA, USA) to further reveal the stress state and distribution in relation to the crack initiation and propagation.

## 3. Results and Discussion

### 3.1. Failure Mode and Fatigue Fracture Characteristics

**Figure 2:**
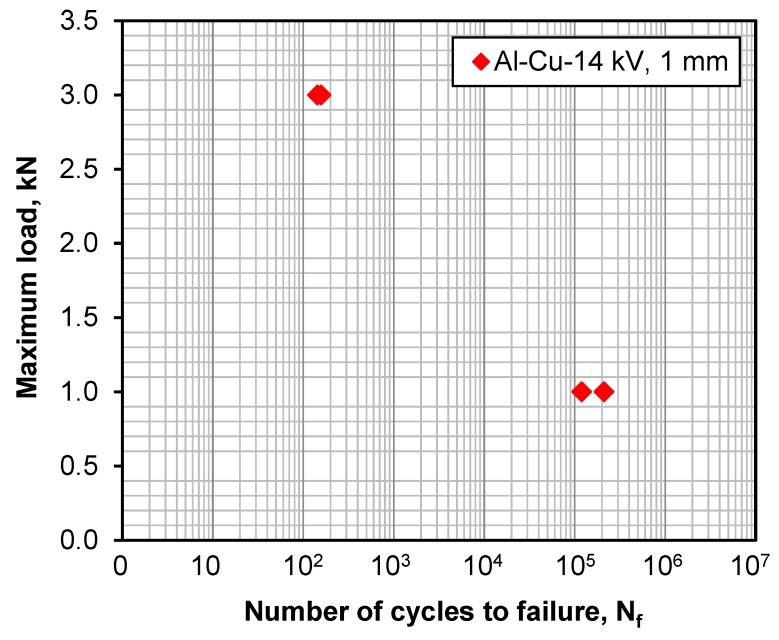
S-N curve of EMPWed Al/Cu dissimilar lap joints fatigued at two loading levels at RT, R = 0.2 and a frequency of 50 Hz.

Fatigue tests at two cyclic loading levels were conducted to examine the failure mode and fatigue fracture characteristics of EMPWed Al/Cu dissimilar lap joints. Considering the experimental scatter in the dynamic fatigue testing, two samples were tested at each loading level. The obtained fatigue life is plotted in Figure 2 in the form of S-N curve.

It is seen that the fatigue data exhibits pretty good reproducibility, especially at a higher loading level of P_max_ = 3 kN. It is normal that a larger experimental scatter could be observed in the fatigue testing at the lower loading level, due to the fact that the long-life/long-time dynamic testing was more susceptible to the presence of potential manufacturing defects, microstructure inhomogeneity, surface roughness, etc.

**Figure 3:**
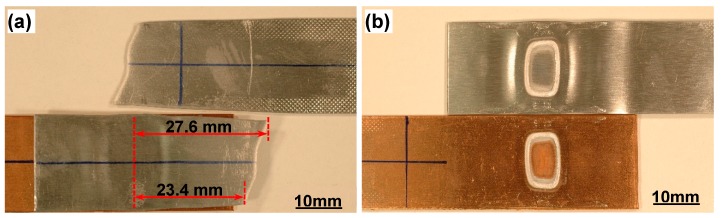
Typical fatigue failure modes of EMPWed Al-Cu lap joints: (**a**) Base metal fracture (BMF) or transverse through-thickness (TTT) crack growth at the maximum cyclic load of 3 kN, and (**b**) Interfacial failure (IF) at the maximum cyclic load of 1 kN.

At the higher loading level of P_max_ = 3 kN failure occurred in the Al base metal (Figure 3a), while interfacial failure occurred at a lower loading level of P_max_ = 1 kN (Figure 3b). Obviously, the failure modes of the dissimilar Al/Cu joints were related to the applied cyclic loading level, the strength of two base metals, and the bonding strength of welded joints. It should be noted that the base metal fracture (BMF) at the higher load is sometimes referred to as transverse through-thickness (TTT) crack growth mode [22,25]. As seen from Figure 3a for a typical BMF sample, failure occurred at a fairly long distance from the weld centerline, i.e., 27.6 mm (upper edge) and 23.4 mm (lower edge) away from welding centerline for this specific sample. This suggests that the bonding strength of the EMPWed Al/Cu dissimilar lap joint was higher than that of Al base alloy in this case. However, the fracture in the base alloy was not the key point in the present study. Figure 3b shows a typical IF sample where failure occurred via shear at the interface, uncovering an annular welding zone on the fracture surface. The IF phenomenon illustrated that yielding and plastic deformation might occur in the bonded region of the EMPWed joint, which would be the focus in this work.

**Figure 4:**
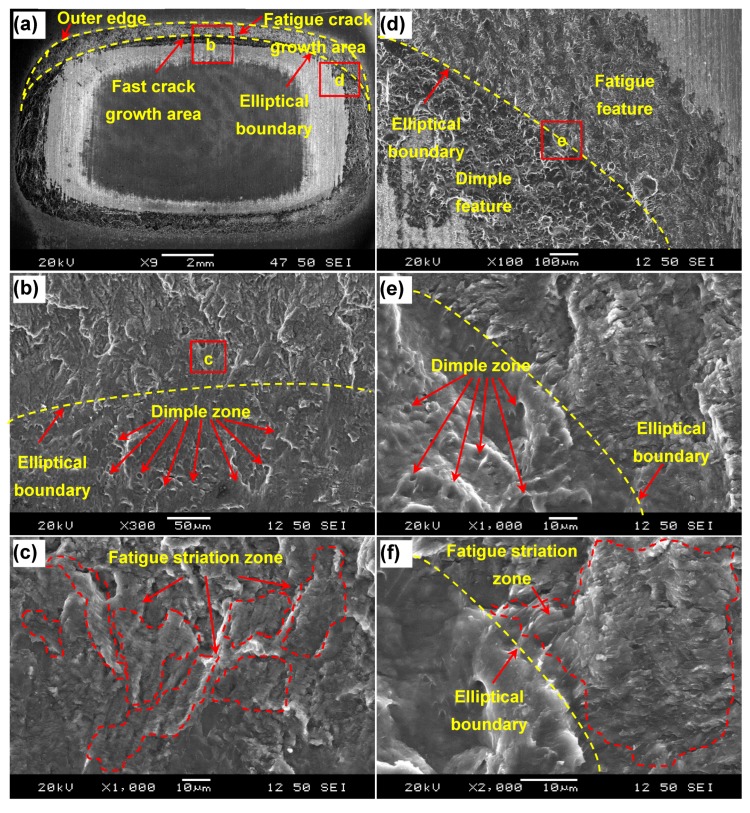
Fracture surface characteristics of an EMPWed Al/Cu dissimilar lap joint fatigued at P_max_ = 1 kN on the Cu side. (**a**) Overall view of the fracture surface; (**b**) typical dimples and elliptical boundary of top side; (**c**) fatigue striation zone of top side; (**d**) fatigue and dimple features and elliptical boundary of right side; (**e**) typical dimple zone of right side, and (**f**) fatigue striation zone of right side.

The typical fracture surface (Cu side) of an Al/Cu dissimilar lap joint tested at P_max_ = 1 kN and failed in the IF mode is shown in Figure 4, where an annular welding zone appeared on the fracture surface. Several features could be identified from an overall view (Figure 4a) and magnified views (Figure 4b–f) of the fracture surface: fatigue crack initiation from the outer edge, a fairly large crack growth area in-between the outer edge and inner elliptical boundary marked by the yellow dashed lines in Figure 4a, and then fast crack growth area. Similar features were observed on the Al side (not shown here). The fast crack growth area was relatively small in comparison with the fatigue crack growth area. The presence of large fatigue crack growth area suggested that yielding and plastic deformation of crack tip occurred during fatigue crack propagation as also observed by others [26,27].The enlarged view for more details is shown in Figure 4c,f for fatigue striations, Figure 4b,e for shear dimples, and for clear elliptical boundary in the upper (Figure 4a) and right (Figure 4d) sides of annular welding zone, respectively. It can be seen that fatigue crack initiated from the outer edge, propagated along the loading direction; as the number of cycles increased, it penetrated through the welding zone towards the inner edge. Mohammed et al. [28] also reported that before the applied stress intensity factor reaches the fracture toughness value of the material in the case of lower cyclic loading level, the crack would grow longer, resulting in a fatigue life mostly spent in the stage of crack propagation. Fatigue crack propagation was characterized by the formation of fatigue striations in the crack propagation zone (Figure 4c,f). It is of interest to note that discrete areas with fatigue striations in short length were widely distributed on the fracture surface as shown in Figure 4c, with a further magnified fatigue striation area shown in Figure 4f. All the special fatigue striations observed above, being roughly perpendicular to the crack propagation direction, underwent the shear load along with a certain degree of opening load due to the presence of bending moment in the tensile lap shear samples. It is known that fatigue striations normally occur via a repeated plastic blunting-sharpening process within the plastic zone in front of fatigue crack tip. This process mainly stemmed from the glide of dislocations on the slip plane along the slip direction. The final fast propagation area consisted of somewhat elongated dimples as indicated by the arrows in Figure 4b,d reflecting the occurrence of final shear fracture in the mode of interfacial failure during fatigue. However, it should be noted that fatigue striations usually occurred in the tensile or opening mode (mode I) fatigue condition, but the fatigue striations described here occurred in the shear mode (mode II) fatigue tests of the EMPWed lap shear samples. To understand this “abnormal” fatigue phenomenon, stress analysis of the Al/Cu lap welded joints has been conducted, along with finite element analysis to reveal the reason why the elliptical boundary appeared in the weld zone.

### 3.2. Stress Analysis and Calculation

**Figure 5:**
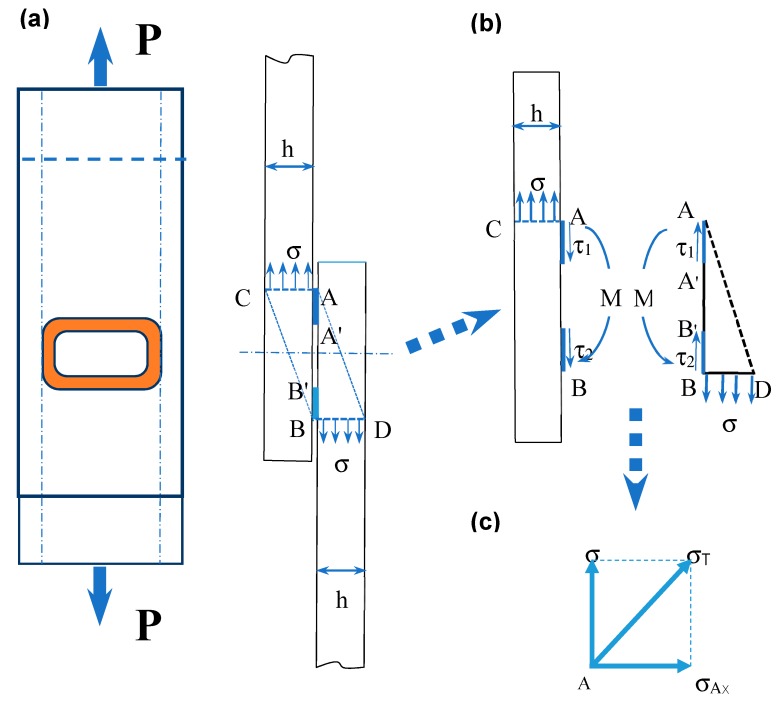
(**a**) Schematic illustration of an EMPWed fatigue (tensile) test specimen, along with (**b**,**c**) stress analysis in the welding zone.

It is well known that the deformation and failure of a tensile lap shear specimen are mainly affected by shear stress in conjunction with a small bending moment. This is attributed to the fact that the force loaded at the end of each side of Al/Cu sample was not in a straight line during fatigue or tensile tests, as shown in Figure 5a. Then fatigue crack initiation and propagation, and the related elliptical boundary and shear dimple formation occurred on the Cu or Al side might be understood from the following analysis. Since the melting point of 6061 Al alloy (with a solidus temperature of ~582 °C) is lower than that of Cu (1085 °C), and the annealed Al alloy was also softer than Cu during joining. Upon EMPW process, a large amount of kinetic energy would be converted to surface deformation energy and adiabatic heating during high-velocity impact. The fast heating and cooling in a few microseconds would lead to a stress concentration and even the potential formation of micro-level cracks at the welding edge. Hence, a further stress analysis for such a failure mode is needed, which is presented in Figure 5. As shown in Figure 5a, an EMPWed specimen under tension is modelled, the welding area is simplified to rectangular annular shape with fillet according to the real shape and size as shown in Figure 4a. Due to the structural misalignment of sheets during the tensile lap shear tests or fatigue tests, a small bending moment *M* should be taken into account together with a tensile force *P* as shown in Figure 5a,b. Therefore, one could easily derive the normal stress (*σ*) and shear stress (*τ*):

(1) $$\sigma =\frac{P}{A_{cross}},$$

(2) $$\tau =\frac{P}{A_{weld}},$$

where *A*_cross_ is the cross-sectional area of base metal, and *A*_weld_ is the annular weld area. The observed failure mode would depend on which one, i.e., the normal stress or shear stress, first reaches its critical value. When the shear stress reaches a critical shear stress (*τ*_o_) in the welding zone, the failure mode would be pure shear, leading to interfacial failure as shown in Figure 4. However, when the shear stress is lower than the critical shear stress, the failure would occur at the site where the stress concentration occurs and the normal stress becomes the maximum which reaches the critical stress (*σ*_o_). It should be noted that in the stress analysis, an area with a unit width (*w* = 1) was used along with some assumptions below:

(1) When A’B’ = 0, the following equations could be obtained according to Equations (1) and (2):

(3) P=σ×h×w=τ×AB×w,

(4) $$\tau =\frac{h}{AB}\sigma .$$

Moreover, a bending moment (*M*) would be present in the fatigue-tensile lap shear testing [29], which may be calculated as:

(5) $$M=\int_{0}^{h}x\sigma wdx=\frac{wh^{2}\sigma }{2}=\frac{h^{2}}{2}\sigma$$

where *h* is the sheet thickness. The maximum shear stress is located on the AB plane, and the shear stress on the AC and BD plane is zero. The maximum tensile stress occurs on the AC or BD plane. The normal stress (*σ_AX_*) resulting from the bending moment (*M*) becomes [30]:

(6) $$\sigma_{AX}=\frac{M\cdot \frac{l}{2}}{I}=\frac{\sigma \cdot \frac{h^{2}}{2}\cdot \frac{l}{2}}{\frac{wl^{2}}{12}}=\frac{3h^{2}}{l^{2}}\sigma ,$$

where *l* = AB, *I* is the moment of inertia. 

As indicated in Figure 5c, If the values of the related stresses are known, one could calculate the principal stress and direction. The total stress (*σ_T_*) at point A becomes the maximum, as shown in Figure 5c, which could be expressed as:

(7) $$\sigma_{T}=\sqrt{\sigma^{2}+\sigma_{AX}{}^{2}}=\sigma \sqrt{1+\frac{9h^{4}}{l^{4}}}.$$

(2) When A’B’ > 0, with an assumption of *τ*_1_ = *τ*_2_ = *τ*, as shown in Figure 5b, Equation (4) could be transformed to Equation (8) below:

(8) $$\tau =\frac{h}{l_{1}+l_{2}}\sigma ,$$

where *l*_1_ = AA’, *l*_2_ = BB’, and the bending moment *M* is the same with Equation (5), then the following equations could be derived:

(9) $$\sigma_{AX}=\frac{M\cdot \frac{l}{2}}{I}=\frac{\sigma \cdot \frac{h^{2}}{2}\cdot \frac{l}{2}}{\frac{wl^{2}}{12}-\frac{wl_{3}^{2}}{12}}=\frac{3h^{2}}{l^{2}(1-{\left(\frac{l_{3}}{l}\right)}^{3})}\sigma ,$$

(10) $$\sigma_{T}=\sqrt{\sigma^{2}+\sigma_{AX}{}^{2}}=\sigma \sqrt{1+\frac{9h^{4}}{l^{4}{\left(1-{\left(\frac{l_{3}}{3}\right)}^{3}\right)}^{2}}},$$

where *l*_3_ = A’B’ as indicted in Figure 5b. Therefore, the failure would first occur at annular welding edge A (or B since the situation of B is equivalent to that of A), in agreement with the experimental observations (i.e., the outer edge shown in Figure 4a).

### 3.3. Finite Element Simulation

**Figure 6:**
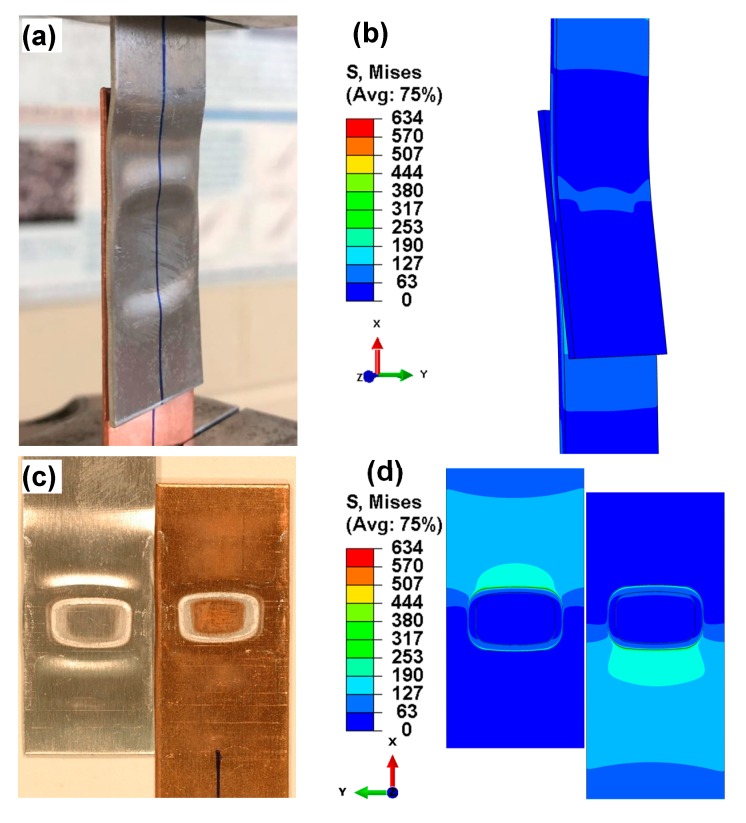
Typical fatigue sample of Al/Cu dissimilar lap joint and FEA simulation results. (**a**) Fatigue test of Al/Cu sample; (**b**) FEA result of (a); (**c**) morphology of fracture surface of Al/Cu sample, and (**d**) FEA result of (c).

The above theoretical stress analysis could reveal the force state and failure location, however, it does not provide sufficient information to understand the failure process and stress distribution. Thus, a 3D stress finite element analysis (FEA) is conducted to further explore the development of stress distribution and validate why elliptical boundary occurred on the fracture surface in the fatigue or tensile test as shown in Figure 4a. To begin, the image of an EMPWed lap joint of Al/Cu sample mounted in the fatigue test machine is shown in Figure 6a, where the sample was fixed by the upper and lower clamps. During fatigue test, cyclic tensile shear loads were applied at the two ends of the joint, with the corresponding boundary conditions through FEM simulation shown in Figure 6b. For simplicity, the following assumptions were made:

(1) The 3D finite element model was obtained based on actual sample sizes and joining condition of the lap welded joint as shown in Figure 1 and Figure 3, via EMPW with weld zones and non-weld zones (Figure 1); the thickness of welding layer and non-weld gap was assumed to be 0.05 mm to perform relatively accurate finite element simulation in the annular welding area.
(2) The lap joint was well bonded with no defects, such as voids and cracks, located at the interface.
(3) All the annular weld areas including transition zone of the joint had the same mechanical properties that were given in Table 3, which were obtained by the average values of the properties of 6061-O Al alloy and Cu.
(4) A tensile load of 1 kN was applied in the finite element analysis to identify the stress distribution and stress concentration location, since the present fatigue loading was all tensile at a stress ratio of R = 0.2.
(5) Clamping indentations were not considered in the finite element model.

Figure 6c shows a typical tensile lap shear fracture surface failed at the applied maximum cyclic load of 1 kN, and Figure 6d presents an overall stress distribution in the annular welding area of fracture surface. It should be noted that finer meshes (a minimum size of local mesh was up to 0.02 mm) were used in the welding area near the edge of the lap weld zone to enhance the precision of calculations, because the location of stress concentration in the theoretical stress analysis was a main concern.

**Figure 7:**
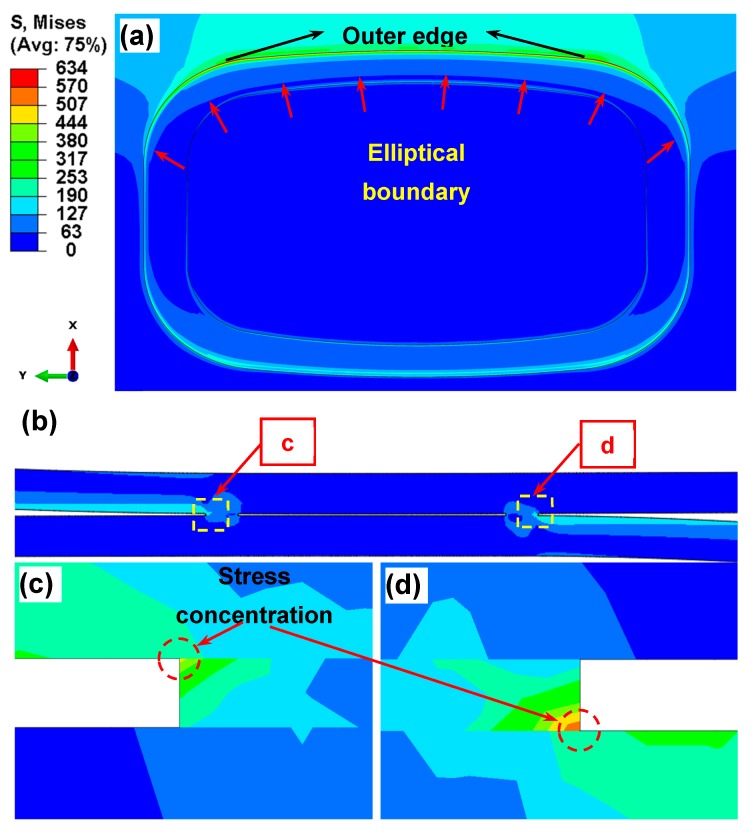
Distribution of von Mises stress and stress concentration area by FEA simulation. (**a**) von Mises stress on the fracture surface; (**b**) deformation and stress concentration zone on the cross section, and a magnified view of the cross-sectional stress concentration zones on the (**c**) Cu side and (**d**) Al side.

The obtained distribution of von Mises stresses (in MPa) on the Al or Cu side (with the same trend of stress state) for the currently used fracture surface of lap welded joint is shown in Figure 7a. The overall view of stress distribution suggests that the maximum stress occurs at the top or bottom region of annular weld area and the stress exhibits a gradient from outer to inner edge. This corresponds to the interfacial failure that would initiate from the outer edge and then undergo crack propagation along the stress gradient direction as the number of cycles increases in the fatigue test. This result coupled with the presence of total stress (*σ*_T_) obtained via Equation (10) would also corroborate the reason for the large fatigue crack propagation area along with the presence of fatigue striations in the welding area as shown in Figure 4. Furthermore, an obvious elliptical stress boundary appears with a changed stress gradient as indicated by the arrow in Figure 7a, which is almost the same as the elliptical dashed curve shown in Figure 4. Therefore, the FEA simulation nicely predicts the unique elliptical boundary that separated the fatigue crack growth area and the fast fracture zone owing to the regularly changing stress gradient during test of lap joints. Figure 7b shows the overall stress distribution across the cross section of EMPWed dissimilar Al/Cu lap joint, with a magnified view shown in Figure 7c,d. It is seen that the stress concentration occurs at interface rooted in the forced outer edge of annular welding area on the Al or Cu side. It follows that crack initiation would occur from these stress concentration sites circled in Figure 7c,d, especially on the Al side where the stress concentration is higher. Therefore, the simulation results obtained from FEA on the EMPWed dissimilar Al/Cu lap joint are in good agreement with the experimental observations and theoretical stress analysis.

## 4. Conclusions

Dissimilar Al 6061-O to Cu-T2 lap joints made with high-speed electromagnetic pulse welding at a discharge voltage of 14 kV and a flying-parent sheet gap of 1 mm have been studied to identify fatigue fracture characteristics including crack initiation and propagation zones in the welding area via fractography, theoretical stress analysis and finite element simulation. The following conclusions can be drawn:

(1) Electromagnetic pulse welding gave rise to an annular (or ring-shaped) bonding area, with the weld zones and a central non-weld zone viewed on the cross section.
(2) Two types of failure modes were observed in relation to the applied cyclic loading levels. Base metal fracture or transverse through-thickness (TTT) crack growth occurred at a higher loading level, while interfacial failure occurred at a lower loading level.
(3) In the interfacial failure, fatigue crack initiated from the outer edge of annular welding area, and propagated from the outer edge to form an approximate elliptical boundary. Fatigue crack propagation was characterized by fatigue striations present in discrete areas on the fracture surface. The final fast fracture started from elliptical boundary with some elongated shear dimples.
(4) Both theoretical stress analysis and finite element model revealed that the maximum stress and stress concentration occurred along the outer edge, in accordance with the experimental observations of fatigue crack initiation site. The theoretical stress analysis also uncovered the existence of a normal stress due to the presence of bending moment caused by the inherent misalignment of a tensile lap shear sample. The combined role of the shear and normal stresses led to the formation of fatigue striations on the fracture surface.

## Figures and Tables

**Table 1:** Chemical composition (wt.%) of the 6061-O Al alloy.

Material	Mg	Si	Cu	Mn	Fe	Zn	Ti	Cr	Ni	Al
6061 O-Al alloy	1.0	0.7	0.3	0.15	0.2	0.25	0.03	0.15	0.05	Bal

**Table 2:** Chemical composition (wt.%) of the Cu-T2.

Material	Bi	Sb	As	Fe	Pb	S	Cu
Copper T2	0.001	0.002	0.002	0.005	0.005	0.005	≥99.90

Note: T2 copper is a Chinese designation for electrolytic tough pitch (ETP) copper, equivalent to UNS C11000 copper.

**Table 3:** Young’s modulus and Poisson ratio of 6061-O Al alloy and Cu-T2.

	Name	6061-O Al Alloy	Copper T2	Annular Shape Welding Area
Properties				
Elastic modulus, *E*(GPa)		69	120	95
Poisson ratio, ν		0.33	0.34	0.33
